# Infection with *Trichomonas vaginalis* increases the risk of psychiatric disorders in women: a nationwide population-based cohort study

**DOI:** 10.1186/s13071-019-3350-x

**Published:** 2019-03-12

**Authors:** Hsin-Chung Lin, Kuo-Yang Huang, Chi-Hsiang Chung, Hsin-An Lin, Rei-Min Chen, Chang-Huei Tsao, Wu-Chien Chien, Tzong-Shi Chiueh

**Affiliations:** 1 Graduate Institute of Medical Sciences, National Defense Medical Center, Taipei, Taiwan; 2 Division of Clinical Pathology, Department of Pathology, Tri-Service General Hospital, National Defense Medical Center, Taipei, Taiwan; 3 Graduate Institute of Pathology and Parasitology, National Defense Medical Center, Taipei, Taiwan; 4 School of Public Health, National Defense Medical Center, Taipei, Taiwan; 5 Taiwanese Injury Prevention and Safety Promotion Association, Taipei, Taiwan; 6 Division of Infection, Department of Medicine, Tri-Service General Hospital SongShan Branch, Taipei, Taiwan; 7 Department of Microbiology and Immunology, National Defense Medical Center, Taipei, Taiwan; 8 Department of Medical Research, Tri-Service General Hospital, National Defense Medical Center, Taipei, Taiwan; 9 Graduate Institute of Life Sciences, National Defense Medical Center, Taipei, Taiwan; 10 Department of Laboratory Medicine, Linkou Chang Gung Memorial Hospital, Taoyuan, Taiwan

**Keywords:** *Trichomonas vaginalis*, Neglected tropical diseases, Psychiatric disorders

## Abstract

**Background:**

*Trichomonas vaginalis* is a protozoan parasite that causes trichomoniasis and annually infects approximately 276 million people worldwide. We observed an ambiguously higher probability of trichomoniasis in patients from the psychiatric department of Tri-Service General Hospital. Herein, we aimed to investigate the association between trichomoniasis and the risk of developing psychiatric disorders.

**Methods:**

The nationwide population-based study utilized the database of the National Health Insurance (NHI) programme in Taiwan. A total of 46,865 subjects were enrolled in this study from 2000–2013, comprising 9373 study subjects with trichomoniasis and 37,492 subjects without trichomoniasis as the control group. Cox proportional hazards regression analysis was performed to calculate the hazard ratio (HR) of psychiatric disorders during the 14 years of follow-up.

**Results:**

Of the study subjects with trichomoniasis, 875 (9.34%) developed psychiatric disorders compared with 1988 (5.30%) in the control group (*P *< 0.001). The adjusted hazard ratio (aHR) of overall psychiatric disorders in the study subjects was 1.644 (95% confidence interval, CI: 1.514–1.766; *P *< 0.001). More specifically, the study subjects had a higher risk for developing an individual psychiatric disorder, including depression, anxiety, bipolar disorder, schizophrenia and substance abuse. Although metronidazole treatment reduced the risk for developing several subgroups of psychiatric disorders, significant reduction was detected for depression only. Furthermore, refractory trichomoniasis (trichomoniasis visits ≥ 2) enhanced the risk of psychiatric disorders.

**Conclusions:**

We show herein that *T. vaginalis* infection increases the overall risk for psychiatric disorders. The novel role of *T. vaginalis* in developing psychiatric disorders deserves more attention, and the control of such a neglected pathogen is of urgent public health importance.

**Electronic supplementary material:**

The online version of this article (10.1186/s13071-019-3350-x) contains supplementary material, which is available to authorized users.

## Background

Human trichomoniasis, caused by *Trichomonas vaginalis*, is the most widespread non-viral sexually transmitted infection, with approximately 276 million cases reported annually worldwide [1]. *Trichomonas vaginalis* infects both women and men, although 89% of trichomoniasis patients are women as a result of their higher occurrence of symptoms [2]. Men are often asymptomatic carriers of *T. vaginalis* infection, although dysuria, discharge and increased risk of infertility and prostate cancer have been reported [3]. Infected women may develop vaginitis, urethritis and cervicitis, potentially leading to serious health outcomes, such as infertility, preterm delivery, low-birth-weight infants, susceptibility to herpes simplex virus and human papillomavirus infection, and cervical cancer [4]. Trichomoniasis has been associated with an increased risk of human immunodeficiency virus (HIV) transmission [5].

In addition to the symptoms and signs, direct microscopic examination, including the wet mount test and Pap smear test, and traditional culture are the most common diagnostic methods for *T. vaginalis* infection. Moreover, rapid antigen detection and nucleic acid amplification test are also used for *T. vaginalis* diagnosis [6].

Current treatments for trichomoniasis include a single oral dose of metronidazole (MTZ; 2 g), a single oral dose of tinidazole (2 g), or a 7-day oral course of MTZ (500 mg twice daily) [7]. The prevalence of trichomoniasis varies among different subpopulations, ranging from 5.4% in family planning clinics and 17.3% in patients presenting to sexually transmitted disease clinics, to 32% among incarcerated women [8, 9]. The prevalence of *T. vaginalis* in women with recurrent urinary tract infections in Taiwan was 16.9% [10]. However, no large-scale epidemiological study of trichomoniasis in Taiwan has been conducted. Hence, it is necessary to understand the prevalence of trichomoniasis for women in Taiwan to improve their sexual and reproductive health.

Psychiatric disorders, also called mental disorders, are defined as clinically significant behavioral or psychological syndromes, with a high level of individual distress, anxiety and premature mortality [11]. In the USA, the regional disease burden attributable to mental disorders, neurological disorders, substance use disorders and self-harm comprises 19% of total disability-adjusted life-years and 34% of total years lived with disability in 2015 [12]. Mental health problems thereby represent important public health challenges worldwide. There is a growing interest in the role of microbes, such as viruses and protozoan parasites, in some psychiatric disorders [13–15]. For instance, several studies have shown impaired cognitive functions among individuals with schizophrenia exposed to neurotropic herpes simplex virus type 1 [16]. Additionally, it has been reported that prenatal maternal exposure to influenza, rubella, genital-reproductive infections and other pathogens are associated with schizophrenia and autism [17, 18]. The protozoan parasite *Toxoplasma gondii* is an extensively studied candidate that is associated with various psychiatric disorders, such as schizophrenia [19, 20]. Having a neurotropic nature and brain-damaging characteristic, *T. gondii* is a potential causative agent for mental and behavioral disorders [14]. However, there is limited evidence for the association of other protists and psychiatric disorders, especially those whose colonization sites are not directly linked to the central nervous system.

Recently, we observed an unexpected trend that trichomoniasis patients were accompanied by some psychiatric disorders in the Tri-Service General Hospital, raising the possibility that there is an association between *T. vaginalis* infection and the risk of psychiatric disorders. Hence, we conducted a nationwide population-based cohort study to verify whether *T. vaginalis* infection may lead to psychiatric disorders. Our findings underscore the potential risk of *T. vaginalis* for developing psychiatric disorders, providing a novel and clinically important role of this neglected protozoan parasite.

## Methods

### Data sources

The National Health Insurance (NHI) programme began in Taiwan in 1995 and covers more than 99% of entire population, with approximately 23 million beneficiaries [21]. The data were collected from the NHI Research Database (NHIRD) of Taiwan. The NHRID uses the International Classification of Diseases, 9th Revision, Clinical Modification (ICD-9-CM) codes to record diagnoses [22]. A subset of the NHIRD, the Longitudinal Health Insurance Database 2000 (LHID 2000), was utilized to investigate the association between trichomoniasis and psychiatric disorders. The LHID 2000 provided a million individuals randomly selected from the entire NHI enrollee population in the year 2000. The ICD-9-CM codes of trichomoniasis-related diagnoses were included in the study group, such as trichomonal vulvovaginitis (ICD-9-CM 131.01), trichomonal urethritis (ICD-9-CM 131.02), other urogenital trichomoniasis (ICD-9-CM 131.09), trichomoniasis of other specified sites (ICD-9-CM 131.8) and unspecified trichomoniasis (ICD-9-CM 131.9). Detailed information of the ICD-9-CM codes used in this study is provided in Additional file 1: Table S1.

### Study design and population

The patients newly diagnosed with trichomoniasis were selected from the LHID 2000 from 1st January 2000 to 31st December 2013. The following criteria were excluded: (i) patients with trichomoniasis before the index date; (ii) patients with psychiatric disorders before tracking; (iii) patients younger than 18 years of age; and (iv) gender is male or unknown. Ultimately, a total of 9373 subjects with trichomoniasis were included in the study group. The non-trichomoniasis control group (37,492 individuals) was established by matching the age and index year with a 4-fold ratio to the study group.

### Covariates

We examined the sociodemographic factors in the study and control groups, including age, monthly income, season, place of residence, urbanization level and hospital level. The patients were classified into three groups based on age: 18–44 years; 45–64 years; and ≥ 65 years. The monthly income in New Taiwan Dollars (NTD) was divided into three groups: <18,000; 18,000–34,999; and ≥35,000. Four seasons (spring, summer, autumn and winter) were considered. The patients living in different areas of Taiwan, including northern, middle, southern, and eastern Taiwan, as well as the outlets islands were compared. The patients were categorized into four urbanization levels from the highest (1) to the lowest (4). Three levels for hospitals where the patients sought medical attention were considered: medical centers; regional hospitals; and local hospitals.

### Main outcome measures

All study participants were followed from the index date until the onset of all recorded psychiatric disorders in the NHIRD. The incidences and risk of each individual psychiatric disorder, including depression, anxiety, bipolar disorder, schizophrenia and substance abuse, were compared between the study group and the control group. The incidences and risk for overall and subgroups of psychiatric disorders in the trichomoniasis patients treated with MTZ were compared with the untreated trichomoniasis patients and the non-trichomoniasis group.

### Statistical analysis

All statistical analyses were performed using SPSS software v.22.0 (SPSS, Chicago, IL, USA). A Chi-square test was used to analyze the categorical variables. Fisher’s exact test was used to evaluate the differences between the study and control groups. Differences in the risk of psychiatric disorders in the study and control groups were evaluated using the Kaplan-Meier method with a log-rank test and presented as a survival curve. Cox proportional hazards regression analysis was used to determine the risk of psychiatric disorder, and the data were expressed as aHR with a 95% confidence interval (CI).

## Results

### Demographic characteristics of the study population at the baseline and endpoint

**Fig. 1:**
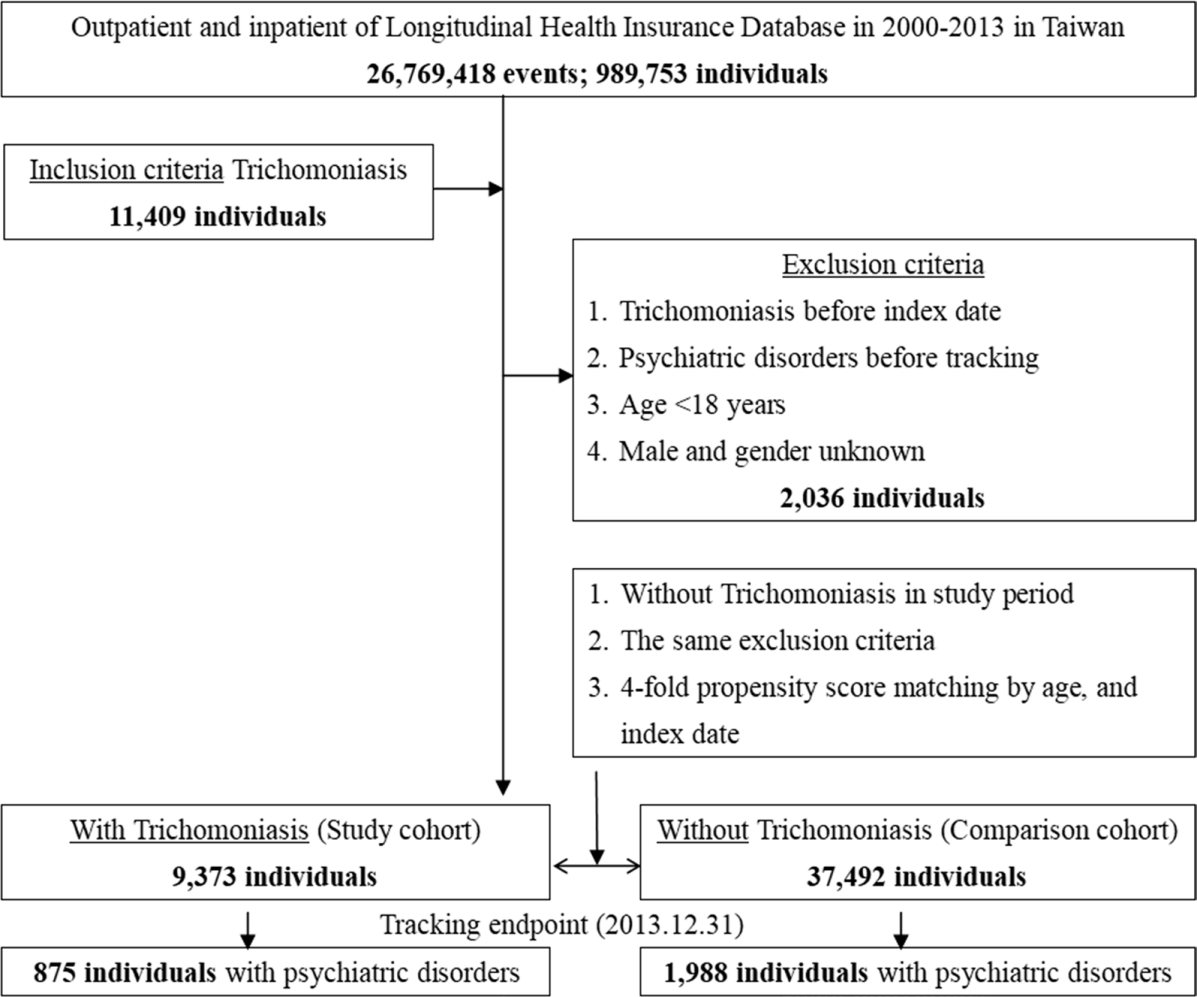
Flowchart of study and control subject’s collection from the Longitudinal Health Insurance Database, a subset of the National Health Insurance Research Database (NHIRD) of Taiwan. The subjects were tracked from 2000 to 2013

Based on propensity score matching (the ratio of the study population to the control population was 1:4), there were 9373 individuals with trichomoniasis in the study group and 37,492 individuals without trichomoniasis in the control group (Fig. 1). The demographic characteristics of the study and control populations at the baseline are described in Table 1. There was no significant difference in age between the control and study groups (42.06 ± 16.09 *vs* 42.09 ± 16.71). The percentage of the population whose monthly income less than NTD $18,000 in the study group was significantly higher than the control group (98.13 *vs* 88.15%; *P* < 0.001). Compared with the control population, the study population had more medical visits in summer (26.51 *vs* 23.95%; *P* < 0.001), with a higher proportion of patients living in eastern Taiwan (14.04 *vs* 4.35%; *P* < 0.001). Regarding the medical care system, more patients with trichomoniasis sought medical help in regional hospitals as compared to the non-trichomoniasis group (49.89 *vs* 29.59%; *P* < 0.001). The demographic characteristics of the study and control populations at the tracking endpoint are described in Additional file 2: Table S2. Except the difference in age between the study and control groups (45.49 ± 19.64 *vs* 46.85 ± 17.85; t-test, *P* < 0.001), all the trends of characteristics between trichomoniasis subjects and non-trichomoniasis subjects were similar to those observed at the baseline.

**Table 1:** Demographic characteristics of the study and control populations at the baseline

Characteristic	Total		With		Without		*P-*value^a^
	*n*	%	*n*	%	*n*	%	
Total	46,865		9373	20.00	37,492	80.00	
Age (years)	42.08 ± 16.59		42.06 ± 16.09		42.09 ± 16.71		0.889
Age group (years)							0.999
18–44	28,350	60.49	5670	60.49	22,680	60.49	
45–64	13,615	29.05	2723	29.05	10,892	29.05	
≥ 65	4900	10.46	980	10.46	3920	10.46	
Insured premium (NT$)							<0.001
< 18,000	42,248	90.15	9198	98.13	33,050	88.15	
18,000–34,999	3221	6.87	154	1.64	3067	8.18	
≥ 35,000	1396	2.98	21	0.22	1375	3.67	
CCI	0.48 ± 1.40		0.61 ± 1.46		0.45 ± 1.39		<0.001
Season							<0.001
Spring (March-May)	12,194	26.02	2380	25.39	9814	26.18	
Summer (June-August)	11,466	24.47	2485	26.51	8981	23.95	
Autumn (September-November)	11,242	23.99	2212	23.60	9030	24.09	
Winter (December-February)	11,963	25.53	2296	24.50	9667	25.78	
Location							<0.001
Northern Taiwan	18,550	39.58	3241	34.58	15,309	40.83	
Middle Taiwan	13,405	28.60	2478	26.44	10,927	29.14	
Southern Taiwan	11,774	25.12	2331	24.87	9443	25.19	
Eastern Taiwan	2947	6.29	1316	14.04	1631	4.35	
Outlets islands	189	0.40	7	0.07	182	0.49	
Urbanization level							<0.001
1 (The highest)	16,142	34.44	2345	25.02	13,797	36.80	
2	20,160	43.02	4746	50.63	15,414	41.11	
3	3815	8.14	630	6.72	3185	8.50	
4 (The lowest)	6748	14.40	1652	17.63	5096	13.59	
Level of care							<0.001
Hospital center	14,329	30.58	2863	30.55	11,466	30.58	
Regional hospital	15,771	33.65	4676	49.89	11,095	29.59	
Local hospital	16,765	35.77	1834	19.57	14,931	39.82	

^a^Chi-square/Fisher’s exact test on categorical variables and t-test on continuous variables

*Abbreviation*: CCI, Charlson comorbidity index

### Association of trichomoniasis with psychiatric disorders

**Fig. 2:**
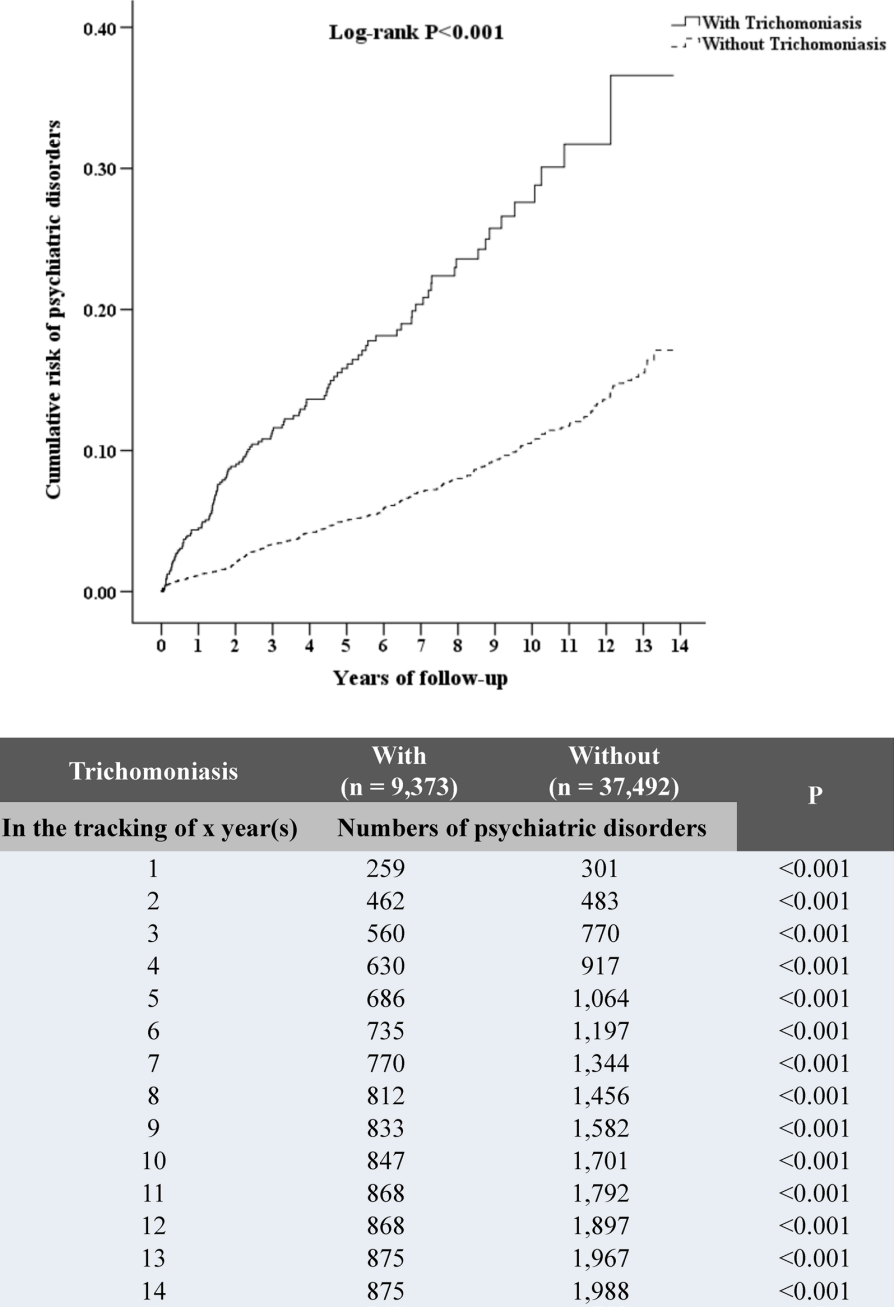
Kaplan-Meier curves for cumulative risk of psychiatric disorders stratified by trichomoniasis with the log-rank test. The numbers of psychiatric disorders in the patients with trichomoniasis and the non-trichomoniasis group are shown during the 14 years of follow-up

The incidences of psychiatric disorders were higher for study subjects with trichomoniasis (875 subjects, 9.34%) than control subjects (1988 subjects, 5.3%) (*P* < 0.001) (Table 2). Additionally, Kaplan-Meier analysis for the cumulative risk of psychiatric disorders during 14 years of follow-up showed a statistical difference in the study group compared with the control group (log-rank *P* < 0.001), and this difference began from the first year of tracking (Fig. 2). The medium duration from the diagnosis of *T. vaginalis* infection to the onset of overall psychiatric disorder was 2.17 years. Additionally, the medium duration from the diagnosis of *T. vaginalis* infection to the onset of individual psychiatric disorder ranged between 0.79–2.34 years (Additional file 3: Table S3). Furthermore, the incidences for the subgroups of the psychiatric disorders were significantly higher in the subjects with trichomoniasis than in the control group, including depression (3.96 *vs* 2.17%; *P* < 0.001), anxiety (3.14 *vs* 1.47%; *P* < 0.001), bipolar disorder (0.45 *vs* 0.28%; *P* = 0.011), schizophrenia (0.97 *vs* 0.39%; *P* < 0.001), substance abuse (0.9 *vs* 0.24%; *P* < 0.001) and other psychiatric disorders (0.82 *vs* 0.15%; *P* < 0.001). The risk of psychiatric disorders in subjects with trichomoniasis was analyzed by Cox regression and presented as adjusted hazard ratio (aHR), with reference to the non-trichomoniasis group (Table 3). The trichomoniasis patients showed a higher risk of overall psychiatric disorders, with an aHR of 1.644 (95% CI: 1.514–1.766; *P* < 0.001).

**Table 2:** Incidence of psychiatric disorders in the trichomoniasis patients compared with the control group

Variable	Total		With		Without		*P*-value^a^
	*n*	%	*n*	%	*n*	%	
Total	46,865		9,373	20.00	37,492	80.00	
Psychiatric disorders							<0.001
Without	44,002	93.89	8498	90.66	35,504	94.70	
With	2863	6.11	875	9.34	1988	5.30	
Depression							<0.001
Without	45,682	97.48	9002	96.04	36,680	97.83	
With	1183	2.52	371	3.96	812	2.17	
Anxiety							<0.001
Without	46,018	98.19	9079	96.86	36,939	98.53	
With	847	1.81	294	3.14	553	1.47	
Bipolar disorders							0.011
Without	46,718	99.69	9331	99.55	37,387	99.72	
With	147	0.31	42	0.45	105	0.28	
PTSD/ASD							0.375
Without	46,816	99.90	9366	99.93	37,450	99.89	
With	49	0.10	7	0.07	42	0.11	
Schizophrenia							<0.001
Without	46,627	99.49	9282	99.03	37,345	99.61	
With	238	0.51	91	0.97	147	0.39	
Substance abuse							<0.001
Without	46,690	99.63	9289	99.10	37,401	99.76	
With	175	0.37	84	0.90	91	0.24	
Other psychiatric disorders							<0.001
Without	46,732	99.72	9296	99.18	37,436	99.85	
With	133	0.28	77	0.82	56	0.15	

^a^Chi-square/Fisher’s exact test on categorical variables and t-test on continuous variables

*Abbreviation*: PTSD, post-traumatic stress disorder; ASD, acute stress disorder

**Table 3:** Risk of psychiatric disorders in the trichomoniasis subjects identified by using Cox regression

Variable	Adjusted HR	95% CI	*P*-value
Without trichomoniasis	Reference		
With trichomoniasis	1.644	1.514–1.766	<0.001

*Abbreviations*: Adjusted HR, adjusted hazard ratio (adjusted for the variables listed in Table 1); CI, confidence interval

### Risk of psychiatric disorders in the trichomoniasis group stratified by covariates

The risk of psychiatric disorders in the trichomoniasis group stratified by variables was further evaluated (Table 4). Except for level of medical care, almost all study subjects kept the higher risk of developing psychiatric disorders irrespective of being stratified by independent variables. Specifically, the trichomoniasis patients stratified by the different age groups revealed that the subjects aged 45–64 years had the highest risk (aHR = 2.637; *P* < 0.001) compared with the non-trichomoniasis control. Additionally, study subjects which had a monthly income of less than NTD $18,000 (aHR = 1.669; *P* < 0.001) were associated with a higher risk of psychiatric disorders. Furthermore, patients with the lowest urbanization level (level 4) (aHR = 3.814; *P* < 0.001) had a markedly increased risk of psychiatric disorders.

**Table 4:** Risk of psychiatric disorders in the trichomoniasis subjects stratified by variables using Cox regression

Stratified	With *vs* without trichomoniasis			
	Adjusted HR	95% CI		*P*-value
Total	1.644	1.514–1.766		<0.001
Age group (years)				
18–44	1.155	1.017–1.312		0.027
45–64	2.637	2.325–2.991		<0.001
≥ 65	0.923	0.696–1.224		0.577
Insured premium (NT$)				
< 18,000	1.669	1.536–1.813		<0.001
18,000–34,999	0.000	–		0.937
≥ 35,000	0.000	–		0.986
Season				
Spring	1.551	1.307–1.841		<0.001
Summer	2.309	2.010–2.651		<0.001
Autumn	1.110	0.906–1.360		0.314
Winter	1.428	1.195–1.706		<0.001
Urbanization level				
1 (the highest)	1.803	1.596–2.037		<0.001
2	1.428	1.209–1.688		<0.001
3	1.291	1.074–1.552		0.007
4 (the lowest)	3.814	2.752–5.285		<0.001
Level of care				
Hospital center	1.479	1.315–1.664		<0.001
Regional hospital	1.594	1.354–1.877		<0.001
Local hospital	2.011	1.693–2.389		<0.001

*Abbreviations*: Adjusted HR, adjusted hazard ratio (adjusted for the variables listed in Table 1); CI, confidence interval; NT$, New Taiwan Dollars

### Reduced risk for the subgroups of psychiatric disorders in the trichomoniasis subjects following MTZ treatment

The risk of the main subgroups of psychiatric disorders in trichomoniasis patients was examined (Table 5). Compared with the non-trichomoniasis group, trichomoniasis subjects had a higher risk of substance abuse (aHR = 2.794; 95% CI: 2.035–3.834; *P* < 0.001), anxiety (aHR = 2.011; 95% CI: 1.738–2.327; *P* < 0.001), schizophrenia (aHR = 1.981; 95% CI: 1.495–2.624; *P* < 0.001), bipolar disorders (aHR = 1.784; 95% CI: 1.241–2.565; *P* < 0.001) and depression (aHR = 1.675; 95% CI: 1.474–1.904; *P* < 0.001). There was no statistical significance in the risk of post-traumatic stress disorder or acute stress disorder (PTSD/ASD) and other psychiatric disorders between the trichomoniasis and non-trichomoniasis groups.

**Table 5:** Risk of psychiatric disorders subgroup in the trichomoniasis patients treated with MTZ identified by using Cox regression

Psychiatric disorder subgroup	Trichomoniasis and metronidazole (MTZ)	Competing risk in the model										
		Population	Event	Adjusted HR	95% CI	*P*	Adjusted HR	95% CI	*P*	Adjusted HR	95% CI	*P*
Overall	Without trichomoniasis	37,492	198	Ref.			Ref.					
	With trichomoniasis	9373	875	1.644	1.514–1.766	<0.001						
	With trichomoniasis, without MTZ	6433	630				1.732	1.577–1.902	<0.001	Ref.		
	With trichomoniasis, with MTZ	2940	245				1.458	1.271–1.672	<0.001	0.876	0.739–1.039	0.128
Depression	Without trichomoniasis	37,492	812	Ref.			Ref.					
	With trichomoniasis	9373	371	1.675	1.474–1.904	<0.001						
	With trichomoniasis, without MTZ	6433	266				1.865	1.613–2.156	<0.001	Ref.		
	With trichomoniasis, with MTZ	2940	105				1.335	1.083–1.647	0.007	0.639	0.493–0.829	0.001
Anxiety	Without trichomoniasis	37,492	553	Ref.			Ref.					
	With trichomoniasis	9373	294	2.011	1.738–2.327	<0.001						
	With trichomoniasis, without MTZ	6433	231				2.120	1.807–2.486	<0.001	Ref.		
	With trichomoniasis, with MTZ	2940	63				1.701	1.301–2.222	<0.001	0.948	0.692–1.300	0.741
Bipolar disorder	Without trichomoniasis	37,492	105	Ref.			Ref.					
	With trichomoniasis	9373	42	1.784	1.241–2.565	0.002						
	With trichomoniasis, without MTZ	6433	35				2.219	1.497–3.287	<0.001	Ref.		
	With trichomoniasis, with MTZ	2940	7				0.908	0.418–1.972	0.807	0.481	0.207–1.118	0.089
PTSD/ASD	Without trichomoniasis	37,492	42	Ref.			Ref.					
	With trichomoniasis	9373	7	0.792	0.354–1.772	0.570						
	With trichomoniasis, without MTZ	6433	7				1.015	0.450–2.288	0.972	Ref.		
	With trichomoniasis, with MTZ	2940	0				0.0001	–	0.963	0.0001	–	0.971
Schizophrenia	Without trichomoniasis	37,492	147	Ref.			Ref.					
	With trichomoniasis	9373	91	1.981	1.495–2.624	<0.001						
	With trichomoniasis, without MTZ	6433	70				2.315	1.699–3.156	<0.001	Ref.		
	With trichomoniasis, with MTZ	2940	21				1.352	0.838–2.180	0.216	0.690	0.385–1.237	0.212
Substance abuse	Without trichomoniasis	37,492	91	Ref.			Ref.					
	With trichomoniasis	9373	84	2.794	2.035–3.834	<0.001						
	With trichomoniasis, without MTZ	6433	56				2.97	2.073–4.256	<0.001	Ref.		
	With trichomoniasis, with MTZ	2940	28				2.516	1.615–3.919	<0.001	0.978	0.428–2.686	0.057
Other psychiatric disorders	Without trichomoniasis	37,492	56	Ref.			Ref.					
	With trichomoniasis	9373	77	1.098	0.872–1.211	0.184						
	With trichomoniasis, without MTZ	6433	49				1.012	0.642–1.19	0.872	Ref.		
	With trichomoniasis, with MTZ	2940	28				1.584	1.104–1.984	0.001	1.971	1.169–3.322	0.011

*Abbreviations*: MTZ, metronidazole; Adjusted HR, adjusted hazard ratio (adjusted for the variables listed in Table 1); CI, confidence interval; PTSD, post-traumatic stress disorder; ASD, acute stress disorder; Ref., reference

The risk for the subgroups of psychiatric disorders in the trichomoniasis subjects treated with MTZ was examined compared with the non-trichomoniasis group and the trichomoniasis subjects without MTZ treatment (Table 5). The trichomoniasis subjects treated with MTZ had a lower risk of developing bipolar disorder and schizophrenia than the trichomoniasis subjects without MTZ treatment, with aHRs of 0.908 (95% CI: 0.418–1.972; *P* = 0.807) and 1.352 (95% CI: 0.838–2.180; *P* = 0.216), respectively, with no statistical difference between the MTZ-treated group and the non-trichomoniasis control. Although MTZ treatment had a lower risk for developing several subgroups of psychiatric disorders with reference to the untreated trichomoniasis subjects, significant reduction was detected for depression only (aHR = 0.639; 95% CI: 0.493–0.829; *P* = 0.001).

### Increased risk of psychiatric disorders in subjects with refractory trichomoniasis

It is estimated that approximately 5–10% of trichomoniasis patients display resistance to drug treatment [23, 24]. We also evaluated the risk for psychiatric disorders in the trichomoniasis subjects who sought medical help more than once (Table 6). Compared with the non-trichomoniasis group, the risk for overall psychiatric disorders in subjects with trichomoniasis was proportional to the number of medical visits. Except for bipolar disorder and PTSD/ASD, refractory trichomoniasis patients (trichomoniasis visits ≥2) had a higher risk for the other psychiatric disorders (*P < *0.001) compared with the patients who sought medical help only once.

**Table 6:** Risk of psychiatric disorders subgroup among study population and trichomoniasis cohort identified by using Cox regression

Psychiatric disorders subgroup	Trichomoniasis visits	Study population					Trichomoniasis cohort		
		Population	Event	Adjusted HR	95% CI	*P*	Adjusted HR	95% CI	*P*
Overall	0 (without trichomoniasis)	37,492	1988	Ref.					
	1 trichomoniasis visit	9051	809	2.560	2.353–2.784	<0.001	Ref.		
	≥ 2 trichomoniasis visits	322	66	7.676	5.953–9.898	<0.001	2.664	2.047–3.467	<0.001
Depression	0	37,492	812	Ref.					
	1	9051	341	2.712	2.381–3.089	<0.001	Ref.		
	≥ 2	322	30	8.306	5.667–12.173	<0.001	2.594	1.741–3.866	<0.001
Anxiety	0	37,492	553	Ref.					
	1	9051	271	2.975	2.562–3.454	<0.001	Ref.		
	≥ 2	322	23	10.674	6.853–16.623	<0.001	3.472	2.199–5.482	<0.001
Bipolar disorder	0	37,492	105	Ref.					
	1	9051	42	2.679	1.854–3.873	<0.001	Ref.		
	≥ 2	322	0	0.000	–	0.968	<0.0001	–	0.972
PTSD/ASD	0	37,492	42	Ref.					
	1	9051	7	1.013	0.456–2.298	0.978	Ref.		
	≥ 2	322	0	0.000	–	0.988	<0.0001	–	0.989
Schizophrenia	0	37,492	147	Ref.					
	1	9051	84	3.949	2.992–5.212	<0.001	Ref.		
	≥ 2	322	7	12.471	5.739–27.101	<0.001	2.806	1.260–6.247	0.012
Substance abuse	0	37,492	91	Ref.					
	1	9051	80	1.535	1.301–2.011	<0.001	Ref.		
	≥ 2	322	4	3.897	2.049–7.122	<0.001	3.286	1.870–5.864	<0.001
Other psychiatric disorders	0	37,492	56	Ref.					
	1	9051	66	1.862	1.503–2.188	<0.001	Ref.		
	≥ 2	322	11	5.897	3.402–9.864	<0.001	5.642	2.864–7.862	<0.001

*Abbreviations*: Adjusted HR, adjusted hazard ratio (adjusted for the variables listed in Table 1); CI, confidence interval; PTSD, post-traumatic stress disorder; ASD, acute stress disorder; Ref., reference

### Association of trichomoniasis with other sexually transmitted infections

It has been shown that *T. vaginalis* infection was associated with other sexually transmitted infections (STIs), such as *Chlamydia trachomatis*, *Neisseria gonorrhoeae*, *Treponema pallidum* and HIV [25–27]. Our data indicated that 3.03% of the subjects with trichomoniasis were co-infected with *N. gonorrhoeae* or *T. pallidum*, 0.61% co-infected with *N. gonorrhoeae* or *C. trachomatis*, and 0.12% co-infected with *T. pallidum* or *C. trachomatis* (Additional file 4: Table S4). Combined analysis revealed that 13.99% of subjects with trichomoniasis were co-infected with one of these three common STIs (*N. gonorrhoeae*, *T. pallidum* or *C. trachomatis*). Additionally, a total of 12 trichomoniasis subjects were infected with HIV, whereas 4 non-trichomoniasis subjects were infected with HIV (Additional file 5: Table S5). Among the trichomoniasis subjects, 5 and 3 subjects were infected with HIV before and after the index date, respectively. For the non-trichomoniasis subjects, 1 and 2 cases were infected with HIV before and after the index date, respectively. All HIV-positive patients were treated.

## Discussion

The trichomoniasis subjects enrolled in this study had a higher risk of overall psychiatric disorders, with an aHR of 1.644, as compared with the non-trichomoniasis control. This means that patients with trichomoniasis had a 1.644-fold increased risk for developing psychiatric disorders. The Kaplan-Meier analysis also supported the cumulative risk for psychiatric disorders in the trichomoniasis subjects during 14 years of follow-up (log-rank *P* < 0.001). More specifically, the trichomoniasis subjects had a significantly increased risk of depression, anxiety, bipolar disorder, schizophrenia and substance abuse. These results highlight the novel role of *T. vaginalis* in causing psychiatric disorders, and that clinicians should pay more attention to the possible risk resulting from this neglected tropical disease.

Previous studies have reported that some psychiatric disorders are associated with inflammatory diseases, such as periodontitis [28], psoriasis [29] and allergic diseases[30]. The underlying mechanism of this association is possibly due to the release of pro-inflammatory cytokines, such as interleukin (IL)-6, IL-10, tumor necrosis factor alpha, and monocyte chemoattractant protein-1, which have been proved to be involved in the development of depression, anxiety and bipolar disorders. *Trichomonas vaginalis* infection has been shown to induce IL-8 secretion from primary human monocytes [31] and the symbiotic relationship with *Mycoplasma hominis* enables induction of an array of inflammatory cytokines in a macrophage cell line [32]. In addition to causing local inflammation, *T. vaginalis* also induces systemic immune response in infected pregnant women, resulting in higher concentrations of granulocyte-macrophage colony-stimulating factor and C-reactive protein in serum of patients [33]. Further investigation is needed to clarify whether the *T. vaginalis*-induced immune response plays a role in developing psychiatric disorders.

Another possibility is that a behavioral pathway may link *T. vaginalis* infection and the risk of psychiatric disorders. For instance, women with *T. vaginalis* infection might present many vaginal symptoms that may affect their sexual life. Their partners might be annoyed with the diagnoses which can complicate their sexual relationship. Therefore, these problems associated with difficulties in getting cured might increase anxiety and other common mental disorders. Additionally, it has been reported that there is an association between high-risk sexual behaviors and sexually transmitted diseases in patients with psychiatric disorders [34]. Thus, the higher *T. vaginalis* infection in psychiatric patients may alternatively have resulted from high-risk sexual behaviors of patients during their prodromal stage.

We found that trichomoniasis subjects aged 45–64 years had a higher risk of psychiatric disorders than those aged 18–44 years. Since the maximal follow-up time is 14 years, we proposed that a certain portion of the trichomoniasis population aged 18–44 years may not reach the age of onset for most major psychiatric disorders [35]. Another possible reason for this observation may be due to the menopausal transition, a period late in a woman’s reproductive life before the final menstruation, typically occurring between the ages of 40 and 55 years. Previous studies demonstrated that women with symptomatic menopausal transition may have a higher risk for developing subsequent psychiatric disorders, especially depression [36], anxiety [37] and bipolar disorder [38], thereby enhancing the risk in the trichomoniasis subjects.

Mental disorders contribute to 7% of the global burden of disease as estimated by disability adjusted life years in the world; this is rising, especially in low- and middle-income countries [39]. Low income has been demonstrated to be directly linked with psychiatric disorders [40]. Indeed, we have revealed that the trichomoniasis subjects with a monthly income less than NTD $18,000 were associated with a higher risk of psychiatric disorders.

Although MTZ treatment for the trichomoniasis patients had a lower risk for developing overall psychiatric disorders, the differences between the treated and untreated groups was not statistically significant. Specifically, MTZ treatment was remarkably associated with a decreased risk of bipolar disorder and schizophrenia, suggesting that *T. vaginalis* infection is closely related with these two psychiatric disorders. Additionally, MTZ treatment was associated with a lower risk for developing depression as compared with the untreated group. Based on these findings, it is likely that trichomoniasis may directly or indirectly involve the process of development for specific psychiatric disorders. Furthermore, increasing reports of failures in the treatment of trichomoniasis and the rising prevalence of MTZ-resistant *T. vaginalis* isolates have occurred [23, 41, 42]. Hence, the differences in treatment outcomes of patients due to drug resistance may also influence the risk for developing psychiatric disorders. Indeed, our further analysis of the trichomoniasis subjects who sought medical help more than once (≥ 2 times) had a higher risk for psychiatric disorders, which is likely caused by drug resistance. Our data revealed that 68.6% patients with trichomoniasis were not treated. A recent study in Belgium demonstrated that 58.1% of women repeatedly positive for *T. vaginalis* infection had received no treatment; this was attributed to low awareness, poor attention, and failure of contact tracing of physicians [43].

One of the major strengths of this study is its large-scale population-based nationwide design. Additionally, the long-term monitoring from 2000 to 2013 made the analysis more reliable. However, the study has several limitations. First, the diagnoses were made using ICD-9 codes recorded in the NHIRD, but this database does not contain all types of data, such as laboratory parameters and genetic factors, which may help to postulate the mechanisms mediating the development of psychiatric disorders in patients with trichomoniasis. Secondly, although men were not included in the study, they potentially transmit the infection to women through sexual behavior and affect the treatment outcomes for women. Hence, sexual partners have to be treated to enhance the treatment efficiency. Thirdly, *T. vaginalis* infection is largely neglected because of ineffective screening protocols and a lack of public health attention [44]. The exact number of patients with trichomoniasis must be higher than those who seek medical attention, and thereby the cases of psychiatric disorders resulted from *T. vaginalis* infection must be underestimated.

## Conclusions

To our knowledge, we provide the first evidence that *T. vaginalis* infection is associated with the risk of overall psychiatric disorders. The potential role of trichomoniasis in the devolvement of psychiatric disorders will highlight its clinical importance and public health impact. Clinicians should pay more attention to this neglected pathogen, which not only results in urogenital symptoms, but also leads to psychiatric disorders, especially in patients with refractory trichomoniasis.

## Additional files

**Additional file 1: Table S1.** ICD-9-CM codes used in this study.

**Additional file 2: Table S2.** Demographic characteristics of the study and control populations at the endpoint.

**Additional file 3: Table S3.** Years to the onset of psychiatric disorders.

**Additional file 4: Table S4.** Sexually transmitted infections of the trichomoniasis cohort.

**Additional file 5: Table S5.** The HIV status of all participants.

## Abbreviations

aHR: adjusted hazard ratio
CI: confidence interval
HR: hazard ratio
HIV: human immunodeficiency virus
IL: interleukin
LHID 2000: Longitudinal Health Insurance Database 2000
MTZ: metronidazole
NHI: National Health Insurance
NHIRD: National Health Insurance Research Database
NTD: New Taiwan Dollars
PTSD: post-traumatic stress disorder
ASD: acute stress disorder
