# Supplementary material for: Infection with Trichomonas vaginalis increases the risk of psychiatric disorders in women: a nationwide population-based cohort study
Source: Parasit Vectors. 2019 Mar 12;12:88. doi: 10.1186/s13071-019-3350-x (PMC6417068; doi:10.1186/s13071-019-3350-x)
Supplement: Supplementary file 1 — Additional file 1: Table S1. ICD-9-CM codes used in this study. [file 13071_2019_3350_MOESM1_ESM.docx]

| **Additional file 1: Table S1. ICD-9-CM codes used in this study** | | |
| --- | --- | --- |
|  | **Abbreviation** | **ICD-9-CM** |
| **Study population:** Trichomoniasis |  | 131 |
| **Event:** Psychiatric disorders |  | 295-312 |
| Depression |  | 296.2-296.3. 300.4, 311 |
| Anxiety |  | 300 |
| Bipolar disorders |  | 296.0, 296.4-296.8 |
| Post-traumatic stress disorder/Acute stress disorder | PTSD/ASD | 308, 309.81 |
| Schizophrenia |  | 295 |
| Substance abuse |  | 303-305 |
| Other psychiatric disorders |  | 295-312, excluding listed above |
