# Supplementary material for: Infection with Trichomonas vaginalis increases the risk of psychiatric disorders in women: a nationwide population-based cohort study
Source: Parasit Vectors. 2019 Mar 12;12:88. doi: 10.1186/s13071-019-3350-x (PMC6417068; doi:10.1186/s13071-019-3350-x)
Supplement: Supplementary file 2 — Additional file 2: Table S2. Demographic characteristics of the study and control populations at the endpoint. [file 13071_2019_3350_MOESM2_ESM.docx]

| **Additional file 2: Table S2. Demographic characteristics of the study and control populations at the endpoint.** | | | | | | | |
| --- | --- | --- | --- | --- | --- | --- | --- |
| **Trichomoniasis** | **Total** | | **With** | | **Without** | | ***P*** |
| **Variables** | **n** | **%** | **n** | **%** | **n** | **%** |  |
| **Total** | 46,865 |  | 9,373 | 20.00 | 37,492 | 80.00 |  |
| **Age (years)** | 46.58 ± 17.68 | | 45.49 ± 19.64 | | 46.85 ± 17.85 | | <0.001 |
| **Age group (years)** |  |  |  |  |  |  | <0.001 |
| 18-44 | 25,256 | 53.89 | 4,963 | 52.95 | 20,293 | 54.13 |  |
| 45-64 | 13,160 | 28.08 | 3,136 | 33.46 | 10,024 | 26.74 |  |
| ≧65 | 8,449 | 18.03 | 1,274 | 13.59 | 7,175 | 19.14 |  |
| **Insured premium (NT$)** |  |  |  |  |  |  | <0.001 |
| <18,000 | 42,248 | 90.15 | 9,198 | 98.13 | 33,050 | 88.15 |  |
| 18,000-34,999 | 3,221 | 6.87 | 154 | 1.64 | 3,067 | 8.18 |  |
| ≧35,000 | 1,396 | 2.98 | 21 | 0.22 | 1,375 | 3.67 |  |
| **CCI** | 0.85 ± 2.44 | | 0.84 ± 2.12 | | 0.86 ± 2.52 | | 0.634 |
| **Season** |  |  |  |  |  |  | <0.001 |
| Spring | 11,746 | 25.06 | 2,191 | 23.38 | 9,555 | 25.49 |  |
| Summer | 11,487 | 24.51 | 2,499 | 26.66 | 8,988 | 23.97 |  |
| Autumn | 12,397 | 26.45 | 2,464 | 26.29 | 9,933 | 26.49 |  |
| Winter | 11,235 | 23.97 | 2,219 | 23.67 | 9,016 | 24.05 |  |
| **Location** |  |  |  |  |  |  | <0.001 |
| Northern Taiwan | 18,697 | 39.90 | 3,276 | 34.95 | 15,421 | 41.13 |  |
| Middle Taiwan | 13,587 | 28.99 | 2,625 | 28.01 | 10,962 | 29.24 |  |
| Southern Taiwan | 11,578 | 24.71 | 2,303 | 24.57 | 9,275 | 24.74 |  |
| Eastern Taiwan | 2,814 | 6.00 | 1,148 | 12.25 | 1,666 | 4.44 |  |
| Outlets islands | 189 | 0.40 | 21 | 0.22 | 168 | 0.45 |  |
| **Urbanization level** |  |  |  |  |  |  | <0.001 |
| 1 (The highest) | 15,673 | 33.44 | 2,422 | 25.84 | 13,251 | 35.34 |  |
| 2 | 20,097 | 42.88 | 4,557 | 48.62 | 15,540 | 41.45 |  |
| 3 | 3,941 | 8.41 | 693 | 7.39 | 3,248 | 8.66 |  |
| 4 (The lowest) | 7,154 | 15.27 | 1,701 | 18.15 | 5,453 | 14.54 |  |
| **Level of care** |  |  |  |  |  |  | <0.001 |
| Hospital center | 14,735 | 31.44 | 2,730 | 29.13 | 12,005 | 32.02 |  |
| Regional hospital | 17,892 | 38.18 | 4,599 | 49.07 | 13,293 | 35.46 |  |
| Local hospital | 14,238 | 30.38 | 2,044 | 21.81 | 12,194 | 32.52 |  |
| *P:* Chi-square / Fisher exact test on category variables and t-test on continue variables; CCI, Charlson comorbidity index. | | | | | | | |
