# Supplementary material for: Infection with Trichomonas vaginalis increases the risk of psychiatric disorders in women: a nationwide population-based cohort study
Source: Parasit Vectors. 2019 Mar 12;12:88. doi: 10.1186/s13071-019-3350-x (PMC6417068; doi:10.1186/s13071-019-3350-x)
Supplement: Supplementary file 3 — Additional file 3: Table S3. Years to the onset of psychiatric disorders. [file 13071_2019_3350_MOESM3_ESM.docx]

**Additional file 3: Table S3. Years to the onset of psychiatric disorders**

| **Psychiatric disorders** | **Overall** | | | |
| --- | --- | --- | --- | --- |
| **Trichomoniasis** | **Min** | **Medium** | **Max** | **Mean ± SD** |
| With | 0.01 | 2.17 | 12.12 | 3.04 ± 2.94 |
| Without | 0.01 | 4.50 | 13.29 | 5.20 ± 3.77 |
| Total | 0.01 | 3.86 | 13.29 | 4.50 ± 3.66 |
| **Psychiatric disorders** | **Anxiety** | | | |
| **Trichomoniasis** | **Min** | **Medium** | **Max** | **Mean ± SD** |
| With | 0.09 | 2.34 | 8.85 | 3.00 ± 2.55 |
| Without | 0.01 | 4.49 | 13.04 | 5.03 ± 3.56 |
| Total | 0.01 | 3.72 | 13.04 | 4.33 ± 3.38 |
| **Psychiatric disorders** | **Depression** | | | |
| **Trichomoniasis** | **Min** | **Medium** | **Max** | **Mean ± SD** |
| With | 0.01 | 2.21 | 12.12 | 3.21 ± 2.94 |
| Without | 0.01 | 4.56 | 13.29 | 5.39 ± 3.82 |
| Total | 0.01 | 3.87 | 13.29 | 4.71 ± 3.71 |
| **Psychiatric disorders** | **Bipolar disorder** | | | |
| **Trichomoniasis** | **Min** | **Medium** | **Max** | **Mean ± SD** |
| With | 0.13 | 0.99 | 3.91 | 1.43 ± 1.34 |
| Without | 0.04 | 5.91 | 11.84 | 5.47 ± 3.75 |
| Total | 0.04 | 3.89 | 11.84 | 4.32 ± 3.72 |
| **Psychiatric disorders** | **PTSD / ASD** | | | |
| **Trichomoniasis** | **Min** | **Medium** | **Max** | **Mean ± SD** |
| With | 0.78 | 0.79 | 1.01 | 0.81 ± 0.99 |
| Without | 0.56 | 2.21 | 7.93 | 3.75 ± 2.95 |
| Total | 0.56 | 2.09 | 7.93 | 3.33 ± 2.92 |
| **Psychiatric disorders** | **Schizophrenia** | | | |
| **Trichomoniasis** | **Min** | **Medium** | **Max** | **Mean ± SD** |
| With | 0.23 | 2.09 | 10.25 | 3.10 ± 3.12 |
| Without | 0.04 | 8.64 | 13.11 | 7.28 ± 3.68 |
| Total | 0.04 | 5.53 | 13.11 | 5.68 ± 4.03 |
| **Psychiatric disorders** | **Substance abuse** | | | |
| **Trichomoniasis** | **Min** | **Medium** | **Max** | **Mean ± SD** |
| With | 0.36 | 1.68 | 10.89 | 3.40 ± 3.47 |
| Without | 0.83 | 3.77 | 12.11 | 5.07 ± 3.37 |
| Total | 0.36 | 3.32 | 12.11 | 4.27 ± 3.51 |
| **Psychiatric disorders** | **Other psychiatric disorders** | | | |
| **Trichomoniasis** | **Min** | **Medium** | **Max** | **Mean ± SD** |
| With | 0.36 | 1.58 | 10.87 | 3.41 ± 3.63 |
| Without | 1.87 | 3.19 | 7.94 | 3.85 ± 2.09 |
| Total | 0.36 | 2.37 | 10.87 | 3.59 ± 3.07 |
| PTSD / ASD, Post-traumatic stress disorder / Acute stress disorder | | | |  |
