# Supplementary material for: Infection with Trichomonas vaginalis increases the risk of psychiatric disorders in women: a nationwide population-based cohort study
Source: Parasit Vectors. 2019 Mar 12;12:88. doi: 10.1186/s13071-019-3350-x (PMC6417068; doi:10.1186/s13071-019-3350-x)
Supplement: Supplementary file 4 — Additional file 4: Table S4. Sexually transmitted infections of the trichomoniasis cohort. [file 13071_2019_3350_MOESM4_ESM.docx]

| **Additional file 4: Table S4. Sexually transmitted infections of Trichomoniasis cohort** | | |
| --- | --- | --- |
| **Sexually transmitted infections** | **n** | **%** |
| Overall | 2,178 | 23.24 |
| *N. gonorrhoeae* only | 204 | 2.18 |
| *T. pallidum* only | 198 | 2.11 |
| *C. trachomatis* only | 113 | 1.21 |
| *N. gonorrhoeae* + *T. pallidum* | 284 | 3.03 |
| *N. gonorrhoeae* + *C. trachomatis* | 57 | 0.61 |
| *T. pallidum* + *C. trachomatis* | 11 | 0.12 |
| *N. gonorrhoeae* + *T. pallidum* + *C. trachomatis* | 1,311 | 13.99 |
| *N. gonorrhoeae* infections | 1,856 | 19.80 |
| *T. pallidum* infections | 1,804 | 19.25 |
| *C. trachomatis* infections | 1,492 | 15.92 |
