# Supplementary material for: Infection with Trichomonas vaginalis increases the risk of psychiatric disorders in women: a nationwide population-based cohort study
Source: Parasit Vectors. 2019 Mar 12;12:88. doi: 10.1186/s13071-019-3350-x (PMC6417068; doi:10.1186/s13071-019-3350-x)
Supplement: Supplementary file 5 — Additional file 5: Table S5. The HIV status of all participants. [file 13071_2019_3350_MOESM5_ESM.docx]

| **Additional file 5: Table S5. The HIV status of all participants** | | |
| --- | --- | --- |
| **Trichomoniasis** | **HIV treatment** | **HIV diagnosed to index date (years)** |
| Without | Yes | -2.76 |
| Without | Yes | 0 |
| Without | Yes | 2.50 |
| Without | Yes | 3.64 |
| With | Yes | -12.08 |
| With | Yes | -3.47 |
| With | Yes | -2.65 |
| With | Yes | -1.01 |
| With | Yes | -0.83 |
| With | Yes | 0 |
| With | Yes | 0 |
| With | Yes | 0 |
| With | Yes | 0 |
| With | Yes | 0.96 |
| With | Yes | 1.42 |
| With | Yes | 1.98 |

HIV, human immunodeficiency virus
